# Prediction of Body Weight of a Person Lying on a Smart Mat in Nonrestraint and Unconsciousness Conditions

**DOI:** 10.3390/s20123485

**Published:** 2020-06-19

**Authors:** Tae-Hwan Kim, Youn-Sik Hong

**Affiliations:** Department of Computer Science and Engineering, Incheon National University, Incheon 22012, Korea; tomxoghks789@inu.ac.kr

**Keywords:** body weight, FSR sensors, smart mat, nonrestraint, unconsciousness

## Abstract

We want to predict body weight while lying in bed for an elderly patient who is unable to move by himself/herself. To this end, we have implemented a prototype system that estimates the body weight of a person lying on a smart mat in nonrestraint and unconsciousness conditions. A total of 128 FSR (force sensing resistor) sensors were placed in a 16 × 8-grid structure on the smart mat. We formulated three methods based on the features to be applied: segmentation, average cumulative sum of pressure, and serialization. All the proposed methods were implemented with four different machine-learning models: regression, deep neural network (DNN), convolutional neural network (CNN), and random forest. We compared their performance using MAE and RMSE as evaluation criteria. From the experimental results, we chose the serialization method with the DNN model as the best model. Despite the limitations of the presence of dead space due to the wide spacing between the sensors and the small dataset, the MAE and the RMSE of the body weight prediction of the proposed method was 4.608 and 5.796, respectively. That is, it showed an average error of ±4.6 kg for the average weight of 72.9 kg.

## 1. Introduction

According to United Nation standards, having 7% of the population aged 65 or older is classified as an aging society and 14% as an aged society [1]. Korea entered the classification of an aged society with the ratio of population aged 65 or older reaching 14.9% in 2019 [2], and the number of elderly people is increasing very rapidly. While the number of facilities such as geriatric hospitals and senior care centers is increasing with this trend, the number of professionals such as nurses and caregivers is insufficient [3].

After the age of 60 years, body weight on average tends to decrease [4]. Recent studies show that low body mass index and weight loss in the elderly are both strong predictors of subsequent mortality [4]. For the same reason, geriatric hospitals and the senior care centers are obliged to measure and record every patient’s weight once a day at a given time to check the patient’s health condition [5].

However, for elderly patients who cannot move by themselves, it is hard to measure their weight using a regular scale. Therefore, caregivers need to either support them or use a special scale to measure their weights. Various methods have been proposed for measuring the weight of a person with impaired mobility. Some were developed as real products, one of which is SECA 684 [6]. It can measure a person’s weight even when he/she is sitting in a wheelchair. However, it is expensive (about $3300) and has the disadvantage of moving a disabled elderly patient into a wheelchair.

The purpose of this paper is to propose a method for predicting the weight of an elderly patient lying in bed within the minimum error range in nonrestraint and unconsciousness conditions. In the latest research, RGB-d sensors [7,8] are used as a method for predicting human weight. The researchers use these sensors to measure anthropometric traits to estimate weight. However, this method requires the subject to be aware of the measurement situation and is sensitive to external environments such as illumination. Another method was proposed to measure weight by wearing smart shoes [9]. This method has a constraint because it uses wearable sensors.

This paper focuses on a method to measure body weight while lying in a bed with a non-wearable sensing method. That is, the target subject is an elderly patient lying in a bed only because he/she cannot move on his/her own. When a person is lying down, the weight is distributed throughout the body. The methods proposed are based the intuitive reasoning that there will be a proportional relationship between one’s weight lying on the smart mat and the cumulative sum of pressures sensed.

Thus, we want to build an IoT (Internet of Things)-based prototype system to measure the weight of a person while lying in bed. In addition, we want to improve the care efficiency by implementing it at a lower price than commercial products. The smart mat cannot only predict one’s weight, but also discriminate one’s lying posture. In our previous study, we published our results to determine lying position using a smart mat [10]. We have added the feature of weight prediction to the existing smart mat’s lying posture discrimination function.

We will present four distinct approaches to predict body weight. Then, each of them will be implemented with four different machine-learning models, i.e., regression, deep neural network (DNN), convolutional neural network (CNN), and random forest, to find the best one with the minimum estimation error. We use the MAE (mean absolute error) and RMSE (root mean square error) as evaluation criteria [11]. Then, we will choose the best model with the minimum MAE (or the minimum RMSE) by comparing all 16 implementations.

The paper is organized as follows: Section 2 describes related works to predict one’s weight using IoT technology. In Section 3, the overall configuration of the smart mat system is explained. Section 4 describes the methods of data collection and the characteristics of the collected data. Section 5 explains the four proposed approaches and compares their performance. Section 6 gives concluding remarks.

## 2. Related Works

Velardo et al. [7] built the model as shown in (1) via multiple regression analysis on a set of anthropometric features, known to be related to human appearance and correlated to weight.

(1) $$weight=-122.27+0.48f_{1}-0.17f_{2}+0.52f_{3}+0.16f_{4}+0.77f_{5}+0.49f_{6}+0.58f_{7}$$

Notice that $f_{1}$, $f_{2}$, $f_{3}$, $f_{4}$, $f_{5}$, $f_{6}$, and $f_{7}$ represent height, upper leg length, calf circumference, upper arm length, upper arm circumference, waist circumference, and upper leg circumference, respectively. To configure the above model, they used the NHANES (National Health and Nutrition Examination Survey) [12] database. This data set was collected from more than 28,000 people. For real case analysis, they took two poses of pictures for 20 subjects using a video surveillance camera. To extract the anthropometric measurements for the objects from the captured images, an estimation of the size of the body parts was computed. The disadvantage of this work is that people have to stand up to take pictures using a camera.

Sazonova et al. [9] made a smart shoe device that uses five force sensing resistor (FSR) sensors embedded in key weight support locations of the insole and three heel-mounted accelerometers. The weight estimation model relies on analysis of pressure measurements during periods of standing with minimal motion. It uses the accelerometer to determine whether the user is in stand mode. To compute features for weight estimation, the mean pressure values (over each 2 s intervals) were computed. After, mean metrics from the left and right shoe for the same sensor location were combined as a simple average, resulting in a total of five metrics (one for each pressure sensor). Notice that they used RMSE as evaluative criteria. Their method is a kind of nonrestraint and unconsciousness approach similar to that aimed at in this paper in that it estimates body weight without requiring additional actions from subjects. However, they have the disadvantage of large prediction error.

Pfitzner et al. [8] present the anthropometric feature extraction from RGB-D (red, green, blue and depth) sensor data, recorded from the frontal view. For weight estimation, people are placed on a stretcher, about 2 m away from the Kinect sensor. The data acquisition was done with the Kinect sensors integrated into the ceiling of a room. To improve the results in body weight estimation, data from the thermal imaging camera was used. Depending on the clothes of a patient, the extracted features differ. Thick clothes result in a higher volume and surface. To minimize this effect the minimum, maximum, and mean temperature of the patient as well as the ambient temperature are extracted and forwarded to the neural network. The disadvantage of their work is that it is affected by environmental factors such as illuminance due to the characteristics of RGB-D sensors [13].

## 3. System Configuration

### 3.1. FSR Sensors

**Figure 1:**
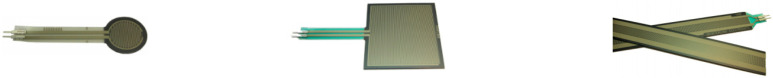
FSR sensors (from left: FSR-402, FSR-406, FSR-408) [15].

A FSR is a special type of resistor whose resistance can be varied by varying the pressure applied to it. If pressure is applied to the surface of sensing film, then the particles touch the conducting electrodes and thus resistance of the film changes [14]. It converts the pressure (0.1 N~10 N) to the corresponding digital value (0~1023). There are three types of FSR sensors depending on size and shape, as shown in Figure 1: round type (FSR-402), square type (FSR-406), and strip type (FSR-408).

The medium size of a medical mattress announced by the Korean Ministry of Food and Drug Safety is 2050 mm × 850 mm [16]. The size of the FSR-402 sensor is 18.27 mm in diameter, so the sensing range is too small to be suitable for such a medical mattress. This is because as the number of sensors increases, the hardware logic controlling them becomes more complicated and thus the power consumption increases. The strip type sensor (e.g., FSR-408) is not suitable for determining the exact position of pressure occurring, since it recognizes pressure when applying force anywhere.

**Figure 2:**
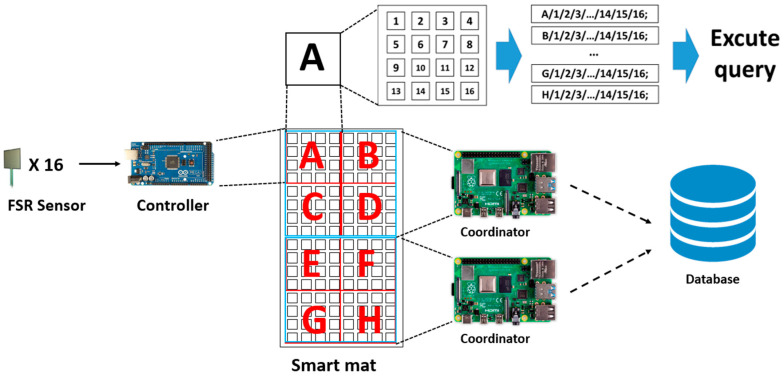
The hardware architecture of the smart mat system.

We have implemented a smart mat using a set of square-shaped FSR-406 sensors with both a width and height of 43.69 mm. It has the widest sensing range among non-strip type sensors. The spacing between them is 63 mm in the vertical direction and 80 mm in the horizontal direction. As shown in Figure 2, the smart mat has 128 FSR sensors placed in the 16 × 8-grid structure. In addition, eight Arduino Mega boards (called controllers) control the whole sensors, where each board is responsible for 16 FSR sensors. Two Raspberry PI boards (called coordinators) control the eight Arduino boards, where each is responsible for four Arduino boards.

### 3.2. The Controller and the Coordinator

As shown in Figure 2, each of the two coordinators is responsible for the upper four controllers (marked from A to D) and the lower four controllers (marked from E to H) of the smart mat, respectively. It was implemented using the Raspberry PI model 3B+ board [17] with a 1.4 GHz 64-bit quad-core processor. It supports dual-band wireless LAN communication. The Raspbian kernel 4.14 packages are installed in it. The four controllers are connected via a USB serial to the corresponding coordinator.

The controller was implemented using an Arduino Mega board [18] with ATmega 2560. The reason for choosing it is that the number of analog I/O pins (=16) is larger than other models, effectively reducing the number of controllers. It receives the values from the corresponding sensors and sends them to the coordinator with its identification. When it transmits the sensed data within a too short period, the amount that the coordinator has to process increases dramatically. Since a person’s lying posture does not change rapidly for a short period, the transmission cycle was set to 2000 ms through experimental analysis.

It is obvious that durability is not completely resolved because the system was implemented as a prototype in the laboratory. However, we have applied two techniques to cope with this problem. As explained earlier, the sensing value of each FSR sensor is sampled at regular intervals, i.e., 2 s. If it is zero consecutively over a certain number of times (set to 10), it is determined that an error has occurred in the corresponding FSR sensor. In addition, as shown in Figure 2, the prototype system has been designed to be as modular as possible using two coordinators and eight controllers to cope with the occurrence of failures. In other words, even if some of the FSR sensors managed by the controller fail, the system will continue to operate with lower accuracy.

As shown in Figure 2, the controller configures the data frame by adding its identification to the sensed data from the 16 FSR sensors it manages and sends it to the coordinator. The coordinator converts the data frame transmitted by the controllers to the corresponding SQL insert statement and transmits it a centralized server via wireless LAN communication. Finally, the server executes the received SQL statement to store the data into the database.

NoSQL (aka not only SQL) databases such as MongoDB are widely used in IoT applications. Rautmare et al. [19] compared the performance of MySQL and MongoDB databases and based the comparison on the time taken to execute Select and Insert queries against varying number of records and threads. They showed that MongoDB required less response time compared to MySQL. However, MySQL responses were more stable compared to MongoDB. Thus, we chose MySQL, focusing on stability rather than response time.

Since the controller is connected to the coordinator via a USB serial, power is supplied to the Arduino board immediately when it is applied to the Raspberry PI board. A battery [20] with a capacity of 20,000 mAh is used as the power supply. The reason why the battery is used as the power source is the mobility. It can be connected to a power outlet for stable power supply.

When the smart mat system starts, the SQL insert statements that insert the sensed data into the database are displayed in log form with the aid of a terminal emulator [21].

## 4. Data Acquisition and Feature Analysis

A reliable large-scale dataset is required to improve the accuracy of weight prediction. In the approach of estimating body weight based on the actual size of a person’s body part, the relevant dataset is relatively abundant [22]. To the best of our knowledge, databases that could fit our needs are not available to the community. Therefore, we have attempted to verify the validity of the proposed methods by utilizing a small dataset for a limited number of subjects.

### 4.1. Data Acquisition

**Figure 3:**
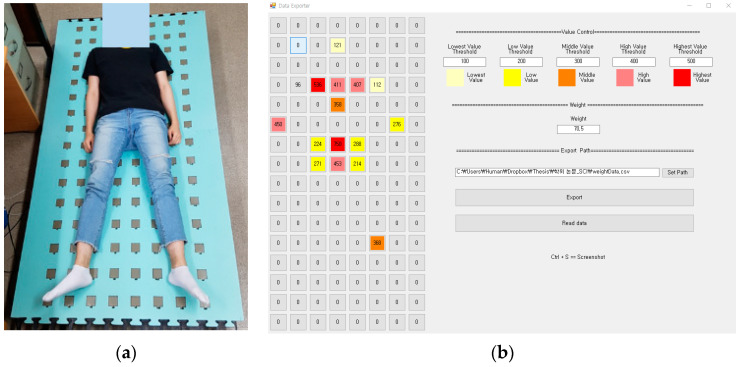
The lying posture in the smart mat (**a**) and the pressure distribution (**b**).

In order to acquire a dataset, a subject lies down in upright posture on the smart mat as shown in Figure 3a. The subject lying down, as shown in Figure 3a, is intended to show the deployment of the FSR sensors in detail. When experimenting at the elderly nursing hospital, the patient lay down with a blanket on the mat so that the FSR sensors did not touch them directly.

Notice that three pressure data were obtained per subject. To avoid sampling bias, the experiment was conducted in such a way that a subject rises up on the mat after acquiring raw data, and then lies down on it again after a few minutes. During this process, the subjects’ hands or feet were not placed outside the area of the mat. Thus, the weight of the subject was distributed within it.

The raw data sensed by the FSR sensors are temporarily saved in the local memory of the coordinator, which is converted into structured data and then stored in the database. A visualization program called “Data Exporter” retrieves the data from the database and displays it, as shown in Figure 3b. It shows the most recently saved data. To increase visibility, it uses different colors from yellow (weak pressure) to red (strong pressure) depending on the pressure value. The reference pressure value that determines the color can be set in the Data Exporter.

Sometimes pressure is measured even though body parts of a subject are not directly in contact with any FSR sensor. This happens because the sensed value was sampled when he/she moved slightly while lying down. After obtaining three consecutive samples of the same subject at 5 s intervals, these data are compared with each other to remove the outlier. We use the GY-100 scale [23] to measure the body weight of a subject to be considered as ground truth. The minimum unit of measurement for this scale is 0.05 kg, with a margin of error of ±0.5 kg.

### 4.2. Feature Extraction

**Figure 4:**
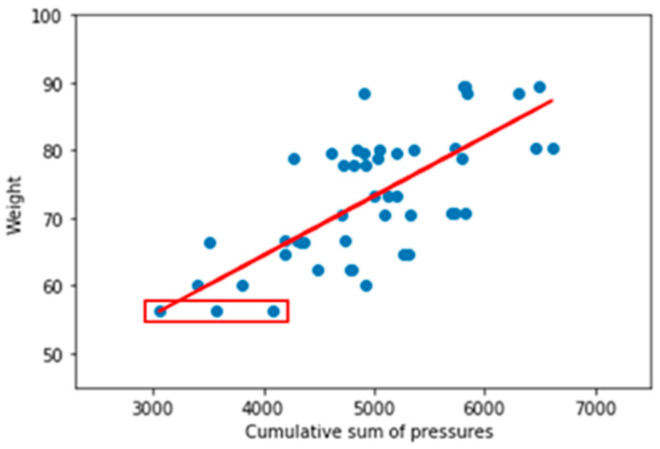
The relationship between body weight and cumulative sum of pressures.

Raw data sampled while the subject is lying on the smart mat is stored in CSV format after removing outliers through pre-processing. The shape of this dataset is 60 samples × 130 columns. The first and second column represent the sampling time and the actual body weight (ground truth), respectively. The remaining 128 columns store all the sensor values sequentially, row by row, starting from the upper left FSR sensor to the lower right FSR sensor. Figure 4 shows the relationship between the subject’s weight and the cumulative sum of pressures. Notice that the range of the weight of the subjects is 56.3~89.4 kg.

**Figure 5:**
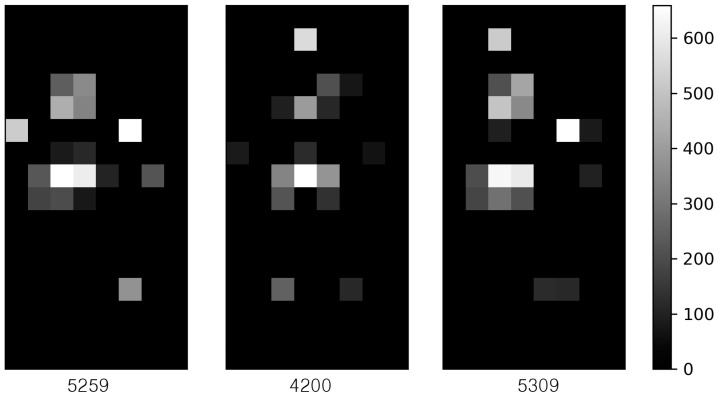
The grey scale image of the three pressure distributions for the same subject.

Figure 5 visualizes the grey-scale image of the pressure distribution of the three samples for the same subject with the weight of 64.8 kg. Notice that the number in Figure 5 represents the cumulative sum of pressures of each sample. The closer it is to white, the more pressure the body part sensed. The subject was guided to lie down in the same position on the smart mat, but the sampling results showed that the pressure distributions were quite different from each other. Thus, the cumulative sum of pressures varies from sample to sample. This is because the spacing between two neighboring FSR sensors is 80 mm in the horizontal direction and 63 mm in the vertical direction, while the sensing range of the FSR sensor is only 45 mm, resulting in a dead zone that cannot be sensed. This is due to the limitations on the number of FSR sensors deployed on the smart mat.

In general, the head, shoulders, and hips are the protruding parts, and the pressure in these body parts are measured more strongly than other parts. In addition, they have a long duration of pressure because of their less movement. Notice that the pressure sensed by arms and legs is weaker than by the head, shoulders, and hips and is not clearly reflected in the pressure distribution. Table 1 summarizes the standard deviation, the average, and the minimum and the maximum values of both the body weight and the cumulative sum of pressures.

## 5. Model Configuration and Performance Evaluation

From the results in Figure 4, we can infer that there is a correlation between the cumulative sum of pressures and weight. Thus, we build a basic method for estimating weight based on the cumulative sum of pressures. Thus, we can formulate it as (2)

(2) $$weight=b+w\times \left(\overset{128}{\underset{i=1}{\sum }}Pressure\left(FSR_{i}\right)\right)$$

Notice that $FSR_{i}$ in (2) refers to the *i*-th FSR sensor. In addition, *b* and *w* represents *bias* and *weight*, respectively.

We measured three cumulative sums of pressures for one subject, and each cumulative sum is considered as a single sample. The weight estimation obtained by applying the basic method will be used as a raw standard for performance evaluation. We propose three other approaches to minimize prediction error.

**Segmentation**: Velardo [7] has proposed a model that uses the lengths and circumferences of the body parts as features. In our preliminary study, we constructed a multiple regression model using three measured values as independent features: sitting height, upper leg circumference, and waist circumference. The accuracy of weight prediction for this model was very high with an MAE of 2.11 kg. For the models to be proposed based on sensed pressure values, the MAE was 4.6 kg~10.3 kg. However, there is the hassle of measuring each body part in advance in order to predict weight. In this study, the cumulative sums of the pressures of the five body parts (head, upper body, lower body, arms, and legs) that can be discriminated based on the sensed pressure are used as the features to predict body weight.

**Average cumulative sum of pressures**: This model predicts weight by using the average cumulative sum of pressures measured three times per subject. When the same person lies on the smart mat, the cumulative sum of pressures is 5259, 4200, and 5390, as shown in Figure 5, with an average of 4949.7 and standard deviation of 652.5. When the average value is used as a training sample, the quality of the measured data will be improved while the size of the dataset is reduced to 1/3.

**Serialization**: This method is to construct a model that uses each of the pressure values sensed by the 128 FSR sensors as an independent feature. At least 75% of the pressure values sensed by the sensors have a value of zero. This will be expected to have a dropout effect.

We want to find the machine-learning model that minimizes the estimation error for the above methods including the basic model. We have implemented each of the four methods by applying four machine-learning models: multiple regression, DNN, CNN, and random forest. In addition, we have used both MAE and RMSE as evaluation criteria.

### 5.1. Experiment Setup

DNN and CNN models have been implemented using Keras [24]. On the other hand, multiple regression and random forest models have been implemented using the scikit-learn library [25]. We applied 10-fold cross-validation to validate the performance of each machine-learning model [26]. That is, performance was compared using the average MAE and the average RMSE for the results of 10 runs.

### 5.2. Model Configuration and Hyperparameter Setting

**Figure 6:**
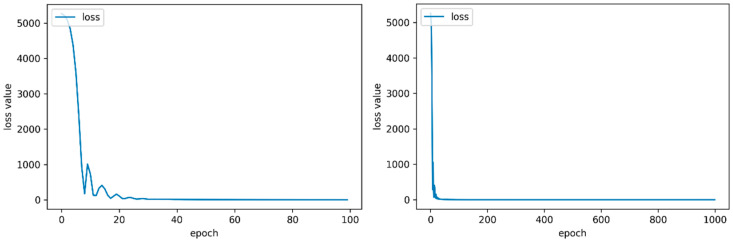
The training loss of the deep neural network (DNN) model with respect to epochs.

The DNN model is composed of an input layer, four hidden layers and an output layer. While training the DNN model, the batch size, one of the hyperparameters, was set to 30 considering the size of the dataset. Epoch was determined to be 150 after analyzing the training loss, as shown in Figure 6. Through the same analysis process, the epoch of the CNN model was set to a larger value (=550) than the DNN.

### 5.3. The Basic Method

With the basic method, we select a linear model of weight with just one attribute, the cumulative sum of pressures. Table 2 summarizes the results of the performance evaluation for the four machine-learning models.

There were no significant differences in performance between these models. Both the regression model and the DNN model showed almost the same performance. For reference purposes, the straight line in Figure 4 is the result of the linear regression model, where the bias and the weight in (2) are 0.0087 and 29.4266, respectively.

### 5.4. Method 1: Segmentation

Based on the pressure distribution sensed by the FSR sensors, the whole body of a person lying on the smart mat is divided into five segments: head, upper body, lower body, arms and legs. The upper body is identified based on the shoulder, and the lower body is identified based on the abdomen and hip. We used multi-tier-based identification algorithms [10] to discriminate the body parts. The cumulative sum $f_{body\text{-}part}$ of pressures corresponding to the recognized body part can be calculated using (3):

(3) $$f_{body\text{-}part}=\sum_{i}pressure\left(FSR_{i}\right),\;\;\mathrm{where}\;FSR_{i}\in body\text{-}part$$

Notice that $pressure\left(FSR_{i}\right)$ represents the pressures sensed by the *i*-th FSR sensor. The model for estimating body weight is configured as (4) using the five cumulative sums as the features:

(4) $$weight=b+w_{1}\times f_{head}+w_{2}\times f_{arms}+w_{3}\times f_{legs}+w_{4}\times f_{upper\text{-}body}+w_{5}\times f_{lower\text{-}body}$$

**Figure 7:**
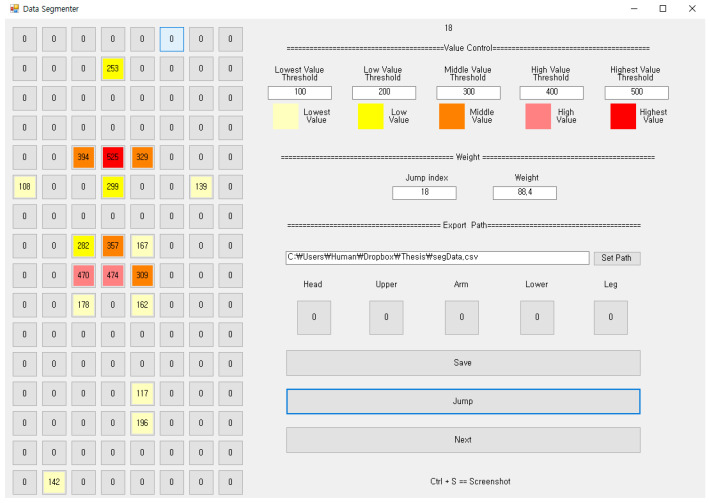
The pressure distribution before segmentation.

**Figure 8:**
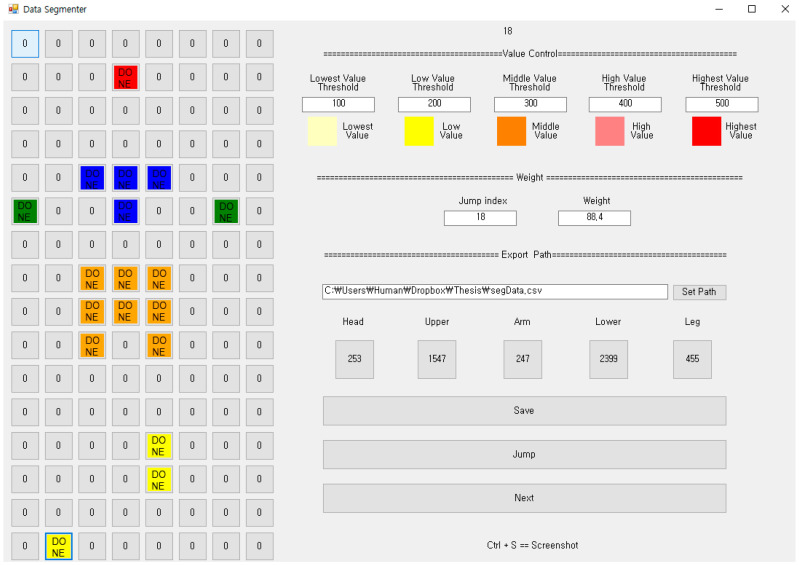
The result of the discrimination of the five body parts.

Figure 7 shows the pressure distribution before segmentation, and Figure 8 shows the result of discrimination of the five body parts. Notice that in Figure 8 the head, the upper body, the lower body, the arm and the leg are represented in red, blue, orange, green and yellow color, respectively. The head, the upper body and the lower body are relatively easy to identify based on pressure distribution. On the other hand, both the arms and legs have experienced frequent recognition errors. This is because, as shown in Figure 7, the pressure on the arms and legs is not only weak, but also occurs remotely like an island.

To compensate for such discrimination errors, we implemented a function of manually designating a specific FSR sensor to one of the body parts. The FSR sensors recognized by the hands and the feet in Figure 8 are the results of manual correction. Notice that the FSR sensors recognized by the hands and the feet are represented in green and yellow colors, respectively.

The hyperparameters were set the same as the basic method. Table 3 summarizes the results of the performance evaluation for the four models using the segmentation method. It shows the improved results compared to the basic method. However, the prediction error was not significantly reduced. The reason why the RMSE is large in this method is that the spacing between neighboring sensors is wide, so that the body parts corresponding to the five features in (4) cannot be properly found.

**Figure 9:**
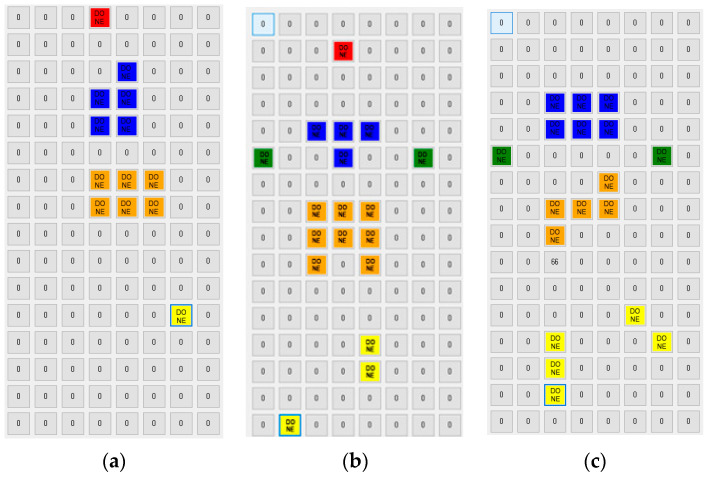
The cases of the incomplete segmentations (**a**,**c**) and the complete segmentation (**b**).

As shown in Figure 9, unlike the center sample, the left sample did not find any arms and legs at all. The pressure on both arms and legs was excluded from the body part recognition process because it was recognized below the threshold (=30). Similarly, the right sample did not distinguish the head at all. When applying the segmentation-based method, only 21 out of the 60 samples were able to recognize the five body parts. We can expect that the performance of this method will be greatly improved if we use a smart mat that has a dense structure by reducing the gap between neighboring sensors to 10 mm.

### 5.5. Method 2: Average Cumulative Sum

This method measures three different samples from one subject and uses the average value of these samples as a single dataset, as shown in (5) or (6):

(5) $$weight=b+w\times \sum_{i=1}^{128}\left(1/3\times \sum_{j=1}^{3}Pressure(FSR_{i}^{j}\right))$$

(6) $$weight=b+1/3\times w\times \sum_{j=1}^{3}\left(\sum_{i=1}^{128}Pressure(FSR_{i}^{j}\right))\;\;\;$$

**Figure 10:**
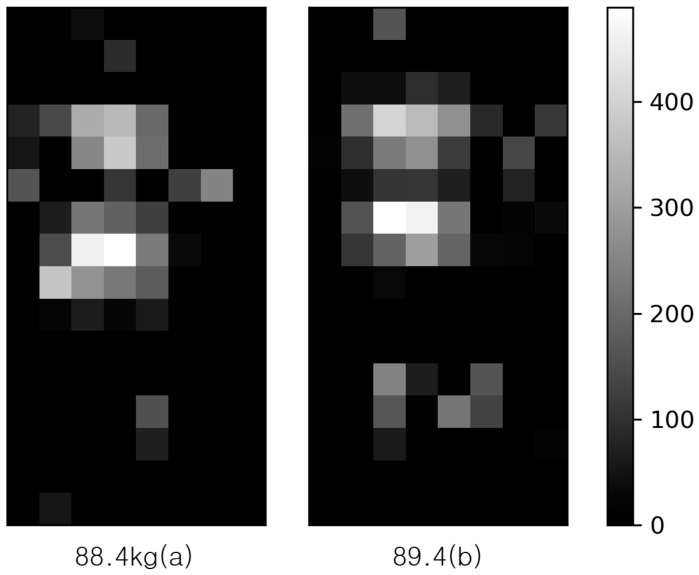
Visualization of the samples using the average sensed value.

Notice that $FSR_{i}^{j}$ is the *j*-th (1≤j≤3) sample sensed by the *i*-th FSR sensor for the same subject. Visualizing the mean of the sensed values makes it relatively easy to identify the pattern of a subject lying down on the smart mat as shown in Figure 10. In Figure 10, the brighter the image, the stronger the pressure intensity. The body weight of the left and the right sample of Figure 10 is 89.4 kg and 88.4 kg, respectively. As explained earlier (in Figure 5), even if a subject lies down while maintaining the same lying posture, the pressure distribution varies with time.

The average cumulative sum method showed a significant increase in both MAE and RMSE compared to the previous two models, as summarized in Table 4. The reason for this is that the size of the dataset has been reduced by 1/3. However, in the case of the CNN model, the input dataset is recognized and processed as an image according to the pressure intensity rather than pressure value, so it shows relatively good results compared to other techniques.

### 5.6. Method 3: Serialization

This method uses the values sensed by all the FSR sensors as independent features: the values from sensor 1 to sensor 128 in order. This can be formulated as (5)

(7) $$weight=b+\sum_{i=1}^{128}\left(w_{i}\times Pressure(FSR_{i}\right))$$

In this method, there is no sensor that can be recognized as an outlier in the preprocessing process. Therefore, all the 128 values sensed using the FSR sensors are included in the training sample. As mentioned earlier, since the value sensed by at least 75% of the 128 FSR sensors is zero or below the threshold, the number of sensors actually reflected in the features is less than 20%. As shown in Table 5, the result of implementing the serialization method using the DNN model is the best.

### 5.7. The Proposed Method: DNN with Serialization

**Figure 11:**
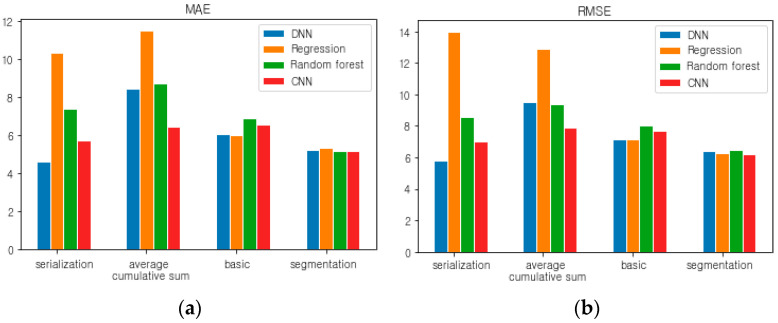
Performance comparison between the four methods with the four learning models (**a**) for the MAE and (**b**) for the RMSE

To estimate the weight of a person lying down on the smart mat, four methods were proposed: basic model, segmentation, average cumulative sum, and serialization. We have compared their performance by implementing each method in various ways with regression, DNN, CNN, and random forest models. The 16 methods were compared based on the MAE and RMSE used as performance indicators, as shown in Figure 11. Notice that the left graph and the right graph in Figure 11 use MAE and RMSE as evaluation criteria, respectively.

**Figure 12:**
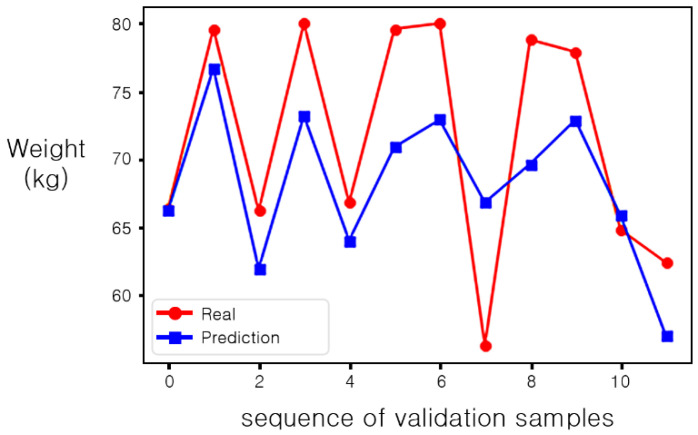
Comparison between the body weights and the predicted values of the proposed model.

Among them, the method of implementing the serialization method with the DNN model yielded the best results (MAE 4.608, RMSE 5.796). Thus, it has been applied to the prediction of the body weight of a person lying on the smart mat and error analysis has been performed based on MAE. In Figure 12, the prediction accuracy was analyzed by applying the serialization-based DNN model to the validation dataset. Notice that in Figure 12 the *x*-axis represents the sequence of validation samples, and the *y*-axis represents a weight (kg).

Table 6 summarizes the body weights (ground truth), the predictions, and the MAEs for the 12 validation samples. The samples with the largest MAE are 6 and 7. For sample 6, the body weight is 81.3 kg, the predicted value is 70.9 kg, and thus the MAE becomes 10.4 kg.

**Figure 13:**
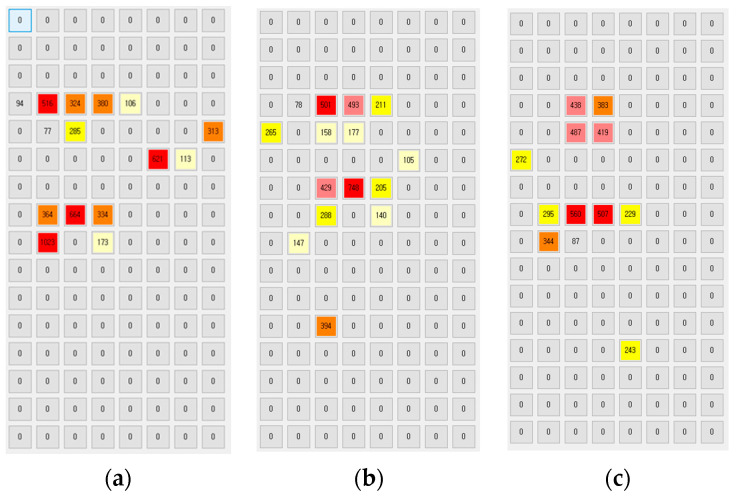
(**a**) The pressure distribution of sample 6 and (**b**,**c**) the pressure distributions of the two samples with the different body weights.

For sample 6, the subject had bent his right arm, as shown in the pressure distribution of Figure 13a, while the other subjects were measured with their arms attached tightly to their bodies, as shown in Figure 3. Due to this, this elbow pressure, which did not occur on the other samples, was sensed and the learning effect was reduced. We defined it as an outlier and re-generated the training dataset with all subjects lying with their arms attached to their bodies. However, the prediction accuracy of other samples except sample 6 was lower than before. Notice that the MAE and the RMSE in this case are 4.879 and 6.264, respectively.

Another case is sample 7 with the body weight of 78.8 kg in Table 6, and the proposed model predicted the sample’s body weight as 69.17 kg, resulting in the MAE of 9.63 kg. The reason for this is that the pressure distribution of sample 5 and sample 7 is similar, as shown in Figure 13b,c. In Figure 13b,c, the actual weight of the two samples is 66.8 kg and 78.8 kg, but the pressure distribution is similar. Notice that the cumulative sum of pressure for the sample in Figure 13b,c is 4261 and 4177, respectively. We think that unexpected recognition errors occurred due to a small dataset.

### 5.8. Performance Comparison between the Proposed Method and Other Methods

**Figure 14:**
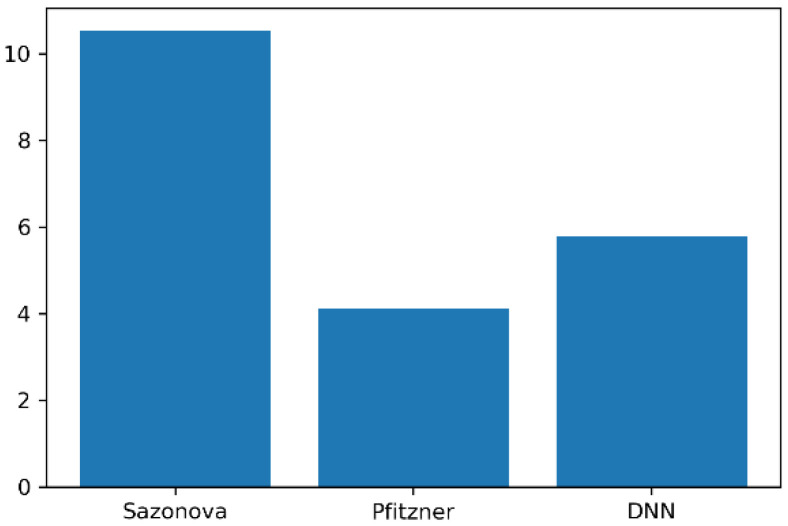
Comparison of performance based on RMSE between Sazonova [9], Pifizner [8], and the proposed models.

Sazonova et al. [9] proposed a method to measure a person’s body weight while standing with smart shoes. The RMSE of the proposed model was 5.912, which shows better performance than the method measured using smart shoes, as shown in Figure 14.

On the other hand, the method in which Pfitzner [8] uses RGB-D sensors to predict body weight has a lower RMSE than the proposed method. Typically, this method using RGB-D sensors has some limitations. The distance they can measure is limited and they suffer from reflection problems on transparent, shiny, or very matte and absorbing objects [13]. In addition, unlike the RGB-D camera-based method, the smart mat system presented in this study has the advantage of not being affected by light.

## 6. Concluding Remarks

In this paper, we implemented a prototype system that measures the body weight of a person lying on the smart mat in nonrestraint and unconsciousness way. A total of 128 FSR-406 sensors are placed in a 16 × 8-grid structure on it. The basic model for predicting body weight of a person lying on it was first implemented using the cumulative sum of pressures sensed by them as a single feature. To improve the performance of this basic method, we proposed three other methods.

The segmentation method uses the five body parts identified through the body discrimination algorithm as features. The average cumulative sum method uses the mean cumulative sum of three samples as a feature to reduce the inherent error of a single sample. The serialization method uses each of the 128 sensors as an independent feature. All the four proposed methods, including the basic method, have been implemented with four different machine-learning models. For all the 16 implementation models, we have compared their performance using MAE and RMSE as performance indicators. From the experimental results, we chose the serialization method with the DNN model as the best model.

There were many dead zones where pressure were not being sensed due to the large horizontal and vertical spacing between neighbor sensors in the smart mat, resulting in more than 75% of all the sensors having a sensing value of zero. This is because the serialization method, which uses valid sensing values as independent features, has an advantage over using the cumulative sum as a single feature. Another reason is that more than 96 of the 128 sensors, which is 75%, have a sensing value of zero, so it may have the dropout effect. On the other hand, since the number of sensors with the non-zero sensing value is relatively small, a recognition error occurred in which two samples with different body weights are classified as the same. This led to an abnormal case that increased the estimated error.

Since the sub array of the FSR sensors responsible for sensing pressures of the lower body does not have a significant effect on the weight prediction, it may be possible to consider how to predict weight using only the sub array of the upper body. However, it is necessary to use complete sensor arrays. This is because the FSR sensors must be placed in a grid so that weight can be measured regardless of the lying direction. It is necessary to estimate the position of the hands and feet to determine the patient’s lying posture. The temporal experiment results showed that the MAE increased by ignoring the measured values of the lower 1/3 of the FSR sensors while predicting body weight. Although 1/3 of the FSR sensors do not measure the values properly, it is difficult to completely ignore them.

The proposed approach has a slightly lower prediction accuracy than the RGB-D sensor-based method. This is because RGB-D sensors have the advantage of additionally acquiring image skeleton and depth information. Nevertheless, the method proposed in this paper has the advantage of being able to measure body weight without any environmental impact. It is expected that improved prediction results can be obtained if the sensors are densely arranged to reduce dead spaces or the size of the dataset is increased.

## Figures and Tables

**Table 1:** The statistical characteristics of the dataset.

Type	Std. Dev.	Avg.	Min.	Max.
weight	9.4	72.9	56.3	89.4
pressures	80.17	4965.75	3055.0	6613.0

**Table 2:** The results of the four models using the basic method.

Model	MAE	RMSE
DNN	6.075	7.131
Regression	6.001	7.130
Random forest	6.873	8.042
CNN	6.526	7.682

**Table 3:** The results for the four models using the segmentation method.

Model	MAE	RMSE
DNN	5.229	6.414
Regression	5.335	6.273
Random forest	5.186	6.495
CNN	5.154	6.195

**Table 4:** The results for the four models using the average cumulative sum method.

Model	MAE	RMSE
DNN	8.435	9.520
Regression	11.517	12.929
Random forest	8.745	9.410
CNN	6.436	7.876

**Table 5:** The results for the four models using the serialization method.

Model	MAE	RMSE
DNN	4.608	5.796
Regression	10.307	14.000
Random forest	7.390	8.590
CNN	5.712	6.990

**Table 6:** Evaluation of the proposed method using validation dataset.

Sequence	Ground Truth	Prediction	MAE
1	66.8	64.535	2.264
2	80.3	77.703	2.596
3	60.2	57.262	2.937
4	62.4	66.499	4.099
5	66.8	69.425	2.625
6	81.3	70.927	10.372
7	78.8	69.169	9.630
8	70.5	66.196	4.303
9	80.0	75.012	4.987
10	71.8	68.537	3.262
11	64.8	66.810	2.010
12	79.6	72.157	7.442
